# Monitoring in Neurointensive Care – The Challenge to Detect Delayed Cerebral Ischemia in High-Grade Aneurysmal SAH

**DOI:** 10.3389/fneur.2014.00134

**Published:** 2014-07-21

**Authors:** Asita S. Sarrafzadeh, Peter Vajkoczy, Philippe Bijlenga, Karl Schaller

**Affiliations:** ^1^Division of Neurosurgery, Geneva Neuroscience Center, Faculty of Medicine, University of Geneva, Geneva, Switzerland; ^2^Department of Neurosurgery, Charité – Universitätsmedizin Berlin, Berlin, Germany

**Keywords:** delayed cerebral ischemia, vasospasm, subarachnoid hemorrhage, aneurysm, neuromonitoring

## Abstract

Delayed cerebral ischemia (DCI) is a feared and significant medical complication following aneurysmal subarachnoid hemorrhage (aSAH). It occurs in about 30% of patients surviving the initial hemorrhage, mostly between days 4 and 10 after aSAH. Clinical deterioration attributable to DCI is a diagnosis of exclusion and especially difficult to diagnose in patients who are comatose or sedated. The latter are typically patients with a high grade on the World Federation of Neurosurgical Societies scale (WFNS grade 4–5), who represent approximately 40–70% of the patient population with ruptured aneurysms. In this group of patients, the incidence of DCI is often underestimated and higher when compared to low WFNS grade patients. To overcome difficulties in diagnosing DCI, which is especially relevant in sedated and comatose patients, the article reports the most recent recommendation for definition of DCI and discusses their advantages and problematic issues in neurocritical care practice. Finally, appropriate neuromonitoring techniques and their clinical impact in high-grade SAH patients are summarized.

## Introduction: The Problem – Delayed Cerebral Ischemia

Delayed cerebral ischemia (DCI) is a feared and significant medical complication following aneurysmal subarachnoid hemorrhage (aSAH). It occurs in about 30% of patients surviving the initial hemorrhage, mostly between days 4 and 10 after aSAH. The known clinical symptoms such as decrease in the level of consciousness and focal signs such as aphasia and hemiparesis may be reversible or otherwise progress to cerebral infarction resulting in an unfavorable outcome or even death (1). Clinical deterioration attributable to DCI is a diagnosis after exclusion of other causes (such as infection, hypotension, hyponatremia, and others), and it is especially difficult to diagnose in patients who are comatose or sedated (1, 2). The latter are typically patients with a high grade on the World Federation of Neurosurgical Societies scale (3) (WFNS grade 4–5), which represent approximately 40–70% of the patient population with ruptured aneurysms. In this group of patients, the incidence of DCI is often underestimated (4) and higher when compared to low WFNS grade patients (5). As shown by Rabinstein and colleagues, as independent predictive factors of a very low risk to develop DCI at age 68 years or older, WFNS I–III at presentation and a modified Fisher grade 1–2 were identified, with specificity of 100% and positive predictive value of 100% (5). As mortality for high-grade patients remains high, efforts have been made to improve diagnosis and treatment of DCI-related damage and to identify a patient subgroup who can expect a better outcome (6).

## DCI and Angiographic Vasospasm

Angiographic vasospasm can be associated with reductions in cerebral perfusion measured with 15O-positron emission tomographic imaging (7). However, regional hypoperfusion and oligemia frequently occurred in territories and patients with no angiographic vasospasm. Furthermore, the development of arterial spasm as diagnosed by angiography or transcranial Doppler (TCD) had no consistent relationship with 3-month outcome (8). Other factors in addition to large-vessel narrowing must contribute to critical reductions in perfusion. So far, because of the common association between clinical deterioration from DCI and angiographic vasospasm, arterial narrowing on angiography and increased blood flow velocities on TCD ultrasound examination are often used as surrogate diagnostic tool (2). This, however, is problematic as the old paradigm of “clinical deterioration due to DCI is equivalent to angiographic vasospasm” has changed in the last years (9). One of the landmarks was the clazosentan-study results (conscious-2), an endothelin-receptor antagonist that effectively resolves angiographic vasospasm, but failed to reduce mortality, DCI-related morbidity, or functional outcome in relation to aSAH (10). Patients with aSAH may have clinical deterioration attributable to DCI in the absence of radiologically confirmed vasospasm (11, 12). This may be the case because angiography is not obtained at the moment of clinical deterioration or because DCI is caused by other factors (2).

## DCI and Infarction – Definitions

Throughout the literature, various means of defining vasospasm are used. To overcome difficulties to diagnose DCI, which is especially relevant in sedated and comatose patients, an excellent summary of possible definitions and their clinical impact, evaluated in 580 aSAH patients, was performed by Frontera and colleagues (8). More recently, to improve comparability of future SAH studies, two different definitions were reformulated by a multidisciplinary research group (2): (1) clinical deterioration caused by DCI and (2) cerebral infarction.

Delayed cerebral ischemia is defined as “the occurrence of focal neurological impairing (such as hemiparesis, aphasia, apraxia, hemianopsia, or neglect) or a decrease of at least two points on the Glasgow Coma Scale [either on the total score or one of its individual components (eye, motor on either side, verbal)]. This should last for at least 1 h, and is not apparent immediately after aneurysm occlusion and cannot be attributed to other causes by means of clinical assessments, cerebral CT or MRI scanning, and appropriate laboratory studies. Nevertheless, the diagnosis of DCI remains fairly subjective.

Cerebral infarction is defined as presence of cerebral infarction on CT or MRI scan of the brain within 6 weeks after SAH, or on the latest CT or MRI scan made before death within 6 weeks, or proven at autopsy, not present on the CT or MR scan between 24 and 48 h after early aneurysm occlusion, and not attributable to other causes (such as clipping or endovascular treatment). Hypodensities on CT imaging resulting from ventricular catheter or intraparenchymal hematoma should not be regarded as cerebral infarctions from DCI.

To remember, cerebral infarction correlates with the territory of angiographic vasospasm in only 25–81% of SAH patients (1, 13). The term “vasospasm” was recommended to be reserved for angiographic arterial narrowing. The occurrence of DCI should be described separately from the results of angiography as not all patients who experience DCI have angiographic vasospasm, and not all patients with angiographic spasm have DCI.

## Real Life in Neurointensive Care

Though knowing that the simple association of radiographic evidence of vasospasm with clinical features of cerebral ischemia does not take into account the different factors that may contribute to DCI, most neurointensivist know a series of cases showing an excellent correlation between an observed clinical deterioration and angiographic narrowing, with deficits easily reversible with an increase of mean arterial blood pressure. In our view, these concern mainly low WFNS grade patients, while in high-WFNS grade patients, the large spectrum of difficult-to-diagnose DCI/infarction is common. The diagnosis of DCI is limited and the diagnosis of cerebral infarction possible only retrospectively once an infarct is found radiographically (8). Obviously, interobserver agreement rate is higher for cerebral infarction and easier to evaluate in high-WFNS grade patients. Nevertheless, a sophisticated evaluation is relevant also in this “difficult to evaluate and treat” patient group, e.g., the clinical course is often complicated by myocardial dysfunction, pneumonia, and acute respiratory distress syndrome secondary to the aneurysm ruptures that further complicate the “vasospasm” treatment and participates in the worst outcome of high WFNS. Some of the factors proceeding to infarction might be reversible if treated. The “infarction diagnosis” reflects an under treatment of ongoing not detected ischemia.

There are a series of worth-to-measure techniques, some of them shortly summarized in the following, aimed to detect “asymptomatic” ischemia in high-WFNS grade patients.

## Possible Causes of DCI

There are other potential mechanisms of primary and secondary brain injury events related to DCI (14), some of them detectable by neuromonitoring techniques.

(1) Intracranial pressure (ICP) and cerebral perfusion pressure (CPP): ICP and CPP monitoring, obviously important components of neurocritical care, are widely used in comatose aSAH patients. ICP is preferentially measured as external ventricular drainage (EVD), while in absence of hydrocephalus/slit ventricles, an ICP probe may be used instead, allowing continuous CPP monitoring. A high ICP (>20 mmHg) is considered as an indirect but unspecific warning sign, is associated with energy failure, high secretion of pro-inflammatory interleukins, and possibly aggravates vasospasm. Optimal CPP is targeted individually depending on several factors (ruptured aneurysm treated and presence of DCI). Since CPP might not be available in all patients, current guidelines (15) recommend (A) maintenance of euvolemia and normal circulating blood volume to prevent DCI (*Class I; Level of Evidence B*, revised recommendation from previous guidelines); (B) (new recommendation) induction of hypertension for patients with DCI unless blood pressure is elevated at baseline or cardiac status precludes it (*Class I*; *Level of Evidence B*); and (C) prophylactic hypervolemia before the development of angiographic spasm is not recommended (*Class III*; *Level of Evidence B*).
(2) Among other pathomechanisms proposed, *ischemia-producing cortical spreading depolarizations* (CSDs) are likely to be involved in DCI development (16). CSDs are monitored by use of subdural electrodes inserted after aneurysm clipping or an EVD. CSD are self-propagating tissue depolarizations, associated with cessation of synaptic activity, surges of extracellular potassium, opening of the blood–brain barrier with edema formation, tissue hypoxia, and inversion to the normal CBF response to neuroglial activity (11, 17). Recent clinical data confirm that a complex cascade of interconnected molecular, metabolic, electrophysiological, and vascular disturbances may lead to ischemia, homologous to animal models (18). The discovery of SDs may lead to a better explanation of DCI in patients with SAH. However, the definite role of SD and its therapeutic implications in SAH are yet unclear (19). Results from the ongoing multicenter Discharge-1 trial (Depolarizations in ISCHaemia after subARachnoid haemorrhaGE-1) are awaited.
(3) *Disturbed cerebrovascular autoregulation* in the first 5 days after SAH significantly increases the odds of DCI occurrence and was shown to have a better predictive value than classical TCD monitoring protocols (20). Initial (first 48 h) preserved cerebrovascular autoregulation in severe SAH grade patients is a good predictor of survival. Optimal CPP (CPPopt) is defined as the lowest CPP corresponding to the strongest cerebrovascular autoregulation activity. It has been shown that CPPopt increases during the vasospasm period for almost all SAH patients but more significantly in the group of patients who suffer vasospasm as assessed by TCDs (21).
(4) *Neurological wake-up test (interruption of sedation, IS-trial)*. Clinical deteriorations, such as occurrence of focal neurological impairing such as aphasia and hemiparesis are warning signs for potentially DCI, which are difficult to evaluate in high-grade SAH patients. The effect of interruption of sedatives and analgesics on neurologic assessment, hemodynamic changes, brain metabolism, and brain tissue oxygen have shown that (1) IS-trials were not attempted on one-third of eligible days due to safety concerns, and that (2) one-third of performed trials had to be stopped due to a critical increase in ICP and impending brain tissue hypoxia. All IS-trials were associated with cardiopulmonary stress, and detection of a new neurological deficit that led to changes in management occurred in only one trial (22). Nevertheless, cerebral metabolism and oxygenation was not affected in a recent trial (traumatic brain injury, TBI, patients) (23). This suggests that IS-trials are safe in the majority of patients but that the test should be individualized and avoided in patients reacting with markedly increased ICP and/or decreased CPP (23). In contrast to TBI, the dynamics for detection of a new deficit in aSAH patients are more variable and potentially treatable, suggesting an important role for IS-trials in aSAH patients. Lacking detailed guidelines, the limitation for this maneuver, in our view, is the stability of ICP and the targeted CPP avoiding arterial hypotension/cerebral hypoperfusion and obviously, ideally under control of further neuromonitoring parameters.
(5) *Monitoring of cerebral oxygenation*. The regionally monitoring brain tissue partial pressure of oxygen sensor and the globally measuring jugular bulb oximetry allowing further analysis of arterial-venous differences of energy-related factors are commonly used techniques for detection of cerebral hypoxia/ischemia. A decrease of brain tissue PO_2_ (<15−10 mmHg) and/or jugular bulb oxygenation (<70%) are warning signs and sensibilize the neurointensivist for occurring processes leading to progressive, clinically not detectable ischemia, infarction, and unfavorable outcome in this high-risk SAH population.
(6) *Cerebral metabolism*. Lacking clinical features to diagnose a neurological deterioration in high-WFNS grade patients, cerebral microdialysis provides information on the metabolic state of the injured brain and allows for determination of a variety of markers of ischemia and immunocompetence in the cerebral extracellular fluid (ECF). This is of relevance since both the blood–brain and blood–CSF barriers may be variably disrupted following aSAH, rendering blood and CSF levels of such markers less reliable. Probes are implanted directly into the region of interest, typically after aneurysm clipping. Profound changes in extracellular glucose, lactate, glutamate, and glycerol in patients with signs of acute or delayed ischemia have been shown (24, 25). Accepted thresholds to indicate metabolic crisis/ischemia are in the first line defined for the lactate–pyruvate ratio >25–40 and glucose <0.5 mM and in second line for glutamate >40 mM; however, individual trends are very important for careful interpretation. The method was especially valuable in patients with acute focal deficits on admission (AFND) due to concomitant intracerebral hemorrhage or edema post aSAH (26, 27). As it is a time- and cost-intense method, only few centers continued monitoring, mainly focusing on possible biomarkers predicting DCI, early brain injury (28), and related inflammatory processes (29).

## Early Brain Injury, DCI, and Immune System

The finding that prevention of delayed vasospasm does not improve outcome in aSAH patients has brought into focus the influence of early brain injury on outcome of aSAH. A substantial amount of evidence indicates that brain injury begins at the aneurysm rupture, evolves with time, and plays an important role in patients’ outcome. It is postulated that intraparenchymal activation of the immune system after aSAH contributes to secondary brain injury. Intraparenchymal inflammation has been linked to progression of aSAH-induced secondary brain damage (30, 31). More specifically, leukocyte–endothelial cell interactions appear to play a critical role in vasospasm. It is hypothesized that the development of DCI is promoted by these inflammatory processes leading to a decreased immunocompetence and secondarily to infectious complications (32). Putative promising markers to indicate immunosuppression are low HLADR and high interleukin-6 levels (thresholds depending on laboratory).

Another point is the entity of patients “at high risk” presenting with acute focal neurological deficits (AFND) on admission (26). Most, but not all of them, are high-WFNS grade patients; however, presenting early brain damages, such as an intracerebral hemorrhage, edema, and high ICP not related to hydrocephalus. These patients show the most severe immunodepression and highest infection rate of the aSAH population (28, 32).

## Summary

In conclusion, the use of clear definitions to classify: (1) occurring deficits such as AFND and DCI, (2) pathomechanisms such as CSD, cerebral hypoxia, disturbed cerebral autoregulation, cerebral metabolism, and inflammation alterations, and (3) resulting lesion using precise time windows and assessment techniques will allow to better share experiences on the disease and improve its understanding. Advanced neuromonitoring techniques may offer the neurointensivists complementary tools to better perceive the pathological condition and engage appropriate treatments that would otherwise lead to progressive, clinically not detectable ischemia, infarction, and unfavorable outcome in high-risk SAH population.

## Conflict of Interest Statement

The authors declare that the research was conducted in the absence of any commercial or financial relationships that could be construed as a potential conflict of interest.

## References

[1] Rabinstein AA Friedman JA Weigand SD Mcclelland RL Fulgham JR Manno EM Predictors of cerebral infarction in aneurysmal subarachnoid hemorrhage. Stroke (2004) 35:1862–6 10.1161/01.STR.0000133132.76983.8e 15218156

[2] Vergouwen MD Vermeulen M van Gijn J Rinkel GJ Wijdicks EF Muizelaar JP Definition of delayed cerebral ischemia after aneurysmal subarachnoid hemorrhage as an outcome event in clinical trials and observational studies: proposal of a multidisciplinary research group. Stroke (2010) 41:2391–5 10.1161/STROKEAHA.110.589275 20798370

[3] Teasdale GM Drake CG Hunt W Kassell N Sano K Pertuiset B A universal subarachnoid hemorrhage scale: report of a committee of the World Federation of Neurosurgical Societies. J Neurol Neurosurg Psychiatry (1988) 51:1457 10.1136/jnnp.51.11.1457 3236024 PMC1032822

[4] Schmidt JM Wartenberg KE Fernandez A Claassen J Rincon F Ostapkovich ND Frequency and clinical impact of asymptomatic cerebral infarction due to vasospasm after subarachnoid hemorrhage. J Neurosurg (2008) 109:1052–9 10.3171/JNS.2008.109.12.1052 19035719

[5] Crobeddu E Mittal MK Dupont S Wijdicks EF Lanzino G Rabinstein AA Predicting the lack of development of delayed cerebral ischemia after aneurysmal subarachnoid hemorrhage. Stroke (2012) 43:697–701 10.1161/STROKEAHA.111.638403 22198987

[6] Hutchinson PJ Power DM Tripathi P Kirkpatrick PJ Outcome from poor grade aneurysmal subarachnoid haemorrhage – which poor grade subarachnoid haemorrhage patients benefit from aneurysm clipping? Br J Neurosurg (2000) 14:105–9 10.1080/02688690050004516 10889881

[7] Dhar R Scalfani MT Blackburn S Zazulia AR Videen T Diringer M Relationship between angiographic vasospasm and regional hypoperfusion in aneurysmal subarachnoid hemorrhage. Stroke (2012) 43:1788–94 10.1161/STROKEAHA.111.646836 22492520 PMC3383942

[8] Frontera JA Fernandez A Schmidt JM Claassen J Wartenberg KE Badjatia N Defining vasospasm after subarachnoid hemorrhage: what is the most clinically relevant definition? Stroke (2009) 40:1963–8 10.1161/STROKEAHA.108.544700 19359629

[9] Pluta RM Hansen-Schwartz J Dreier J Vajkoczy P Macdonald RL Nishizawa S Cerebral vasospasm following subarachnoid hemorrhage: time for a new world of thought. Neurol Res (2009) 31:151–8 10.1179/174313209X393564 19298755 PMC2706525

[10] Macdonald RL Higashida RT Keller E Mayer SA Molyneux A Raabe A Clazosentan, an endothelin receptor antagonist, in patients with aneurysmal subarachnoid haemorrhage undergoing surgical clipping: a randomised, double-blind, placebo-controlled phase 3 trial (CONSCIOUS-2). Lancet Neurol (2011) 10:618–25 10.1016/S1474-4422(11)70108-9 21640651

[11] Ostergaard L Aamand R Karabegovic S Tietze A Blicher JU Mikkelsen IK The role of the microcirculation in delayed cerebral ischemia and chronic degenerative changes after subarachnoid hemorrhage. J Cereb Blood Flow Metab (2013) 33:1825–37 10.1038/jcbfm.2013.173 24064495 PMC3851911

[12] Woitzik J Dreier JP Hecht N Fiss I Sandow N Major S Delayed cerebral ischemia and spreading depolarization in absence of angiographic vasospasm after subarachnoid hemorrhage. J Cereb Blood Flow Metab (2012) 32:203–12 10.1038/jcbfm.2011.169 22146193 PMC3272613

[13] Weidauer S Lanfermann H Raabe A Zanella F Seifert V Beck J Impairment of cerebral perfusion and infarct patterns attributable to vasospasm after aneurysmal subarachnoid hemorrhage: a prospective MRI and DSA study. Stroke (2007) 38:1831–6 10.1161/STROKEAHA.106.477976 17446425

[14] Sabri M Lass E Macdonald RL Early brain injury: a common mechanism in subarachnoid hemorrhage and global cerebral ischemia. Stroke Res Treat (2013) 2013:394036 10.1155/2013/394036 23533958 PMC3603523

[15] Sander Connolly E Rabinstein AA Carhuapoma JC Derdeyn CP Dion J Higashida RT Guidelines for the management of aneurysmal subarachnoid hemorrhage: a guideline. Stroke (2012) 43:1711–37 10.1161/STR.0b013e3182587839 22556195

[16] Dreier JP The role of spreading depression, spreading depolarization and spreading ischemia in neurological disease. Nat Med (2011) 17:439–47 10.1038/nm.2333 21475241

[17] Dreier JP Major S Pannek HW Woitzik J Scheel M Wiesenthal D Spreading convulsions, spreading depolarization and epileptogenesis in human cerebral cortex. Brain (2012) 135:259–75 10.1093/brain/awr303 22120143 PMC3267981

[18] Sakowitz OW Santos E Nagel A Krajewski KL Hertle DN Vajkoczy P Clusters of spreading depolarizations are associated with disturbed cerebral metabolism in patients with aneurysmal subarachnoid hemorrhage. Stroke (2013) 44:220–3 10.1161/STROKEAHA.112.672352 23223504

[19] Sanchez-Porras R Zheng Z Santos E Scholl M Unterberg AW Sakowitz OW The role of spreading depolarization in subarachnoid hemorrhage. Eur J Neurol (2013) 20:1121–7 10.1111/ene.12139 23551588

[20] Budohoski KP Czosnyka M Smielewski P Kasprowicz M Helmy A Bulters D Impairment of cerebral autoregulation predicts delayed cerebral ischemia after subarachnoid hemorrhage: a prospective observational study. Stroke (2012) 43:3230–7 10.1161/STROKEAHA.112.669788 23150652

[21] Bijlenga P Czosnyka M Budohoski KP Soehle M Pickard JD Kirkpatrick PJ “Optimal cerebral perfusion pressure” in poor grade patients after subarachnoid hemorrhage. Neurocrit Care (2010) 13:17–23 10.1007/s12028-010-9362-1 20405341

[22] Helbok R Kurtz P Schmidt JM Stuart MR Fernandez L Connolly SE Effects of the neurological wake-up test on clinical examination, intracranial pressure, brain metabolism and brain tissue oxygenation in severely brain-injured patients. Crit Care (2012) 16:R226 10.1186/cc11880 23186037 PMC3672610

[23] Skoglund K Hillered L Purins K Tsitsopoulos PP Flygt J Engquist H The neurological wake-up test does not alter cerebral energy metabolism and oxygenation in patients with severe traumatic brain injury. Neurocrit Care (2014) 20(3):413–26 10.1007/s12028-013-9876-4 23934408

[24] Nilsson OG Brandt L Ungerstedt U Saveland H Bedside detection of brain ischemia using intracerebral microdialysis: subarachnoid hemorrhage and delayed ischemic deterioration. Neurosurgery (1999) 45:1176–84; discussion 1184–5. 10.1097/00006123-199911000-00032 10549935

[25] Sarrafzadeh A Haux D Plotkin M Ludemann L Amthauer H Unterberg A Bedside microdialysis reflects dysfunction of cerebral energy metabolism in patients with aneurysmal subarachnoid hemorrhage as confirmed by 15 O-H2 O-PET and 18 F-FDG-PET. J Neuroradiol (2005) 32:348–51 10.1016/S0150-9861(05)83168-2 16424838

[26] Sarrafzadeh A Haux D Sakowitz O Benndorf G Herzog H Kuechler I Acute focal neurological deficits in aneurysmal subarachnoid hemorrhage: relation of clinical course, CT findings, and metabolite abnormalities monitored with bedside microdialysis. Stroke (2003) 34:1382–8 10.1161/01.STR.0000074036.97859.02 12750537

[27] Sarrafzadeh A Haux D Kuchler I Lanksch WR Unterberg AW Poor-grade aneurysmal subarachnoid hemorrhage: relationship of cerebral metabolism to outcome. J Neurosurg (2004) 100:400–6 10.3171/jns.2004.100.3.0400 15035274

[28] Radolf S Smoll N Drenckhahn C Dreier JP Vajkoczy P Sarrafzadeh AS Cerebral lactate correlates with early onset pneumonia after aneurysmal SAH. Transl Stroke Res (2014) 5:278–85 10.1007/s12975-013-0292-z 24323715

[29] Sarrafzadeh A Santos E Wiesenthal D Martus P Vajkoczy P Oehmchen M Cerebral glucose and spreading depolarization in patients with aneurysmal subarachnoid hemorrhage. Acta Neurochir Suppl (2013) 115:143–7 10.1007/978-3-7091-1192-5_28 22890660

[30] Sehba FA Pluta RM Zhang JH Metamorphosis of subarachnoid hemorrhage research: from delayed vasospasm to early brain injury. Mol Neurobiol (2011) 43:27–40 10.1007/s12035-010-8155-z 21161614 PMC3023855

[31] Sehba FA Bederson JB Nitric oxide in early brain injury after subarachnoid hemorrhage. Acta Neurochir Suppl (2011) 110:99–103 10.1007/978-3-7091-0353-1_18 21116923

[32] Sarrafzadeh A Schlenk F Meisel A Dreier J Vajkoczy P Meisel C Immunodepression after aneurysmal subarachnoid hemorrhage. Stroke (2011) 42:53–8 10.1161/STROKEAHA.110.594705 21088239

